# Linoleic Acid: A Narrative Review of the Effects of Increased Intake in the Standard American Diet and Associations with Chronic Disease

**DOI:** 10.3390/nu15143129

**Published:** 2023-07-13

**Authors:** Joseph Mercola, Christopher R. D’Adamo

**Affiliations:** 1 Natural Health Partners, LLC, 125 SW 3rd Place, Cape Coral, FL 33991, USA; 2 Department of Family and Community Medicine, Center for Integrative Medicine, University of Maryland School of Medicine, Baltimore, MD 21201, USA; cdadamo@som.umaryland.edu

**Keywords:** linoleic acid (LA), seed oils, cardiolipin, oxidized linoleic acid metabolites (OXLAMs), 4-hydroxynonenal (HNE), omega-3, omega-6

## Abstract

The intake of linoleic acid (LA) has increased dramatically in the standard American diet. LA is generally promoted as supporting human health, but there exists controversy regarding whether the amount of LA currently consumed in the standard American diet supports human health. The goal of this narrative review is to explore the mechanisms that underlie the hypothesis that excessive LA intake may harm human health. While LA is considered to be an essential fatty acid and support health when consumed in modest amounts, an excessive intake of LA leads to the formation of oxidized linoleic acid metabolites (OXLAMs), impairments in mitochondrial function through suboptimal cardiolipin composition, and likely contributes to many chronic diseases that became an epidemic in the 20th century, and whose prevalence continues to increase. The standard American diet comprises 14 to 25 times more omega-6 fatty acids than omega-3 fatty acids, with the majority of omega-6 intake coming from LA. As LA consumption increases, the potential for OXLAM formation also increases. OXLAMs have been associated with various illnesses, including cardiovascular disease, cancer, and Alzheimer’s disease, among others. Lowering dietary LA intake can help reduce the production and accumulation of OXLAMs implicated in chronic diseases. While there are other problematic components in the standard American diet, the half-life of LA is approximately two years, which means the damage can be far more persistent than other dietary factors, and the impact of reducing excessive LA intake takes time. Therefore, additional research-evaluating approaches to reduce OXLAM formation and cardiolipin derangements following LA consumption are warranted.

## 1. Introduction

Polyunsaturated fatty acids (PUFAs) are basic components involved in the architecture and function of cellular membranes and play key roles in several biological processes. PUFAs are endogenous mediators for cell signaling and are involved in the regulation of gene expression. They are also metabolic precursors of eicosanoids, such as prostaglandins and leukotrienes, and docosanoids, such as protectins or resolvins [1].

The most common source of PUFAs in the human diet today are vegetable and seed oils that contain linoleic acid (LA), which is an omega-6 fatty acid. LA is a major constituent of human tissues [2], and it is considered to be an essential fatty acid. Deficiencies in essential fatty acid intake among adults have been documented for decades. [3,4] Furthermore, the important effects of LA in animal and human physiology have been studied for decades [5,6,7,8,9], and a modest, evolutionarily consistent intake of LA has been associated with a decreased risk of atherosclerosis [10], hypercholesterolemia [11,12], headaches when combined with omega-3 fatty acid supplementation [13], and other chronic health conditions.

However, when the levels of LA become highly elevated in the blood through dietary intake in amounts that far exceed recommended amounts, this PUFA becomes a precursor to oxidized LA metabolites (OXLAMs), such as 4-Hydroxynonenal (HNE), 9- and 13-hydroxy-octadecadienoic acid (9- and 13-HODE), and 9- and 13-oxo-octadecadienoic acid (9- and 13-oxoODE). OXLAMs are discussed in depth in a subsequent section of this review.

Furthermore, LA conversion may lead to the formation of free radicals, such as 8-hydroxyoctanoic acid and heptanoic acid [14]. In addition, in some instances, LA may be further metabolized into arachidonic acid (AA), which is a precursor to oxidized AA metabolites (OXAAMs), including 5-, 8-, 9-, 11-, 12-, and 15-hydroxy-eicosatetraenoic acid (HETE) [15]. The increased circulation of oxidized metabolites and free radicals has been linked to different types of diseases (e.g., cardiovascular, atherosclerotic, hepatic, etc.) [16].

LA intake has become increasingly controversial considering its widespread promotion as a healthy fat despite a growing body of evidence suggesting that the current consumption, being exponentially higher than historical norms, may be harmful and a contributor to chronic diseases. This narrative review evaluates the evidence that gave rise to the consideration of LA as an essential fatty acid, the mechanisms of activity of LA in the human diet, and the hypothesis that there is a deleterious impact on human health when LA is consumed in amounts that are typical in the standard American diet today, far exceeding recommended amounts.

## 2. How Much LA Is Required in the Human Diet?

The first demonstration of the essential requirement for LA in animal diets was obtained by Burr and Burr in 1929–1930 [13]. They showed that rats receiving 0.6% of their total dietary calories as LA were 30% higher in body weight compared to total-fat-deficient rats and did not develop skin desquamation and tail necrosis [17,18,19].

This led to the establishment of the requirement of 1% of the total daily calories being omega-6 fat, which was later extended to 2% of the total daily calorie intake in humans to ensure sufficiency. This was confirmed by two studies where the physiological symptoms of omega-6 deficiency in human infants, as established by scaling of the skin, were abolished [20].

However, a careful review of the data used to establish LA being essential to the diet found that this conclusion was established using control diets that were not only deficient in omega-6 fatty acids, but also omega-3 fatty acids. This dual deficiency in the control diets seemed to invalidate the establishment of an omega-6 fatty acid requirement from these data [21].

Subsequent studies in rat models similar to those used to originally classify LA as an essential fatty acid have demonstrated that the dietary omega-3 fatty acid, alpha-linolenic acid (ALA), is able to diminish the symptoms of LA deficiency [22]. This strongly suggests that the absence of ALA in the original studies probably heightened the significance of the physiological symptoms caused by LA deficiency. It seems that, at least for the rat model, the nutritional requirement for LA has probably been seriously overestimated. A more precise estimation of the LA requirement is likely closer to a 75% reduction, or 0.5%, of the dietary energy rather than 2% [23].

This calls into question how “essential” LA really is in the human diet, especially since, outside of a research lab or parenteral nutrition, it is virtually impossible to avoid ingesting enough LA to meet physiological needs in diets across the world today. Currently, most American adults are consuming far more than the recommended amounts of LA. According to the Institute of Medicine (IOM), the dietary guidelines for LA intake recommend an upper limit of 10% [24], which is much higher than the optimal level of 1–2% [25]. Despite the higher than optimal IOM dietary guidelines, the United States Department of Agriculture (USDA) reports that most adults are still consuming far above that limit [26].

## 3. Presence of LA in Human Breast Milk and Relevance to Human Nutrition

LA is present in varying amounts in human breast milk. A robust evidence base extending back decades revealed that the amount of LA in breast milk is largely dependent upon the amount of LA that the mother consumes and the presence of LA in maternal adipose tissue.

A seminal study conducted in 1959 [27] provided lactating women with a high LA diet consisting of fats from lard, corn oil, or linseed oil, which approximated the LA content of the standard American diet, with approximately 15–30% of total calories coming from LA. Within 2–3 days of changing their usual diet to the high LA diet, the amounts of LA in their breast milk increased from 8–10% to 42%. The study highlights that the LA content of breast milk has significantly increased in human breast milk due primarily to marked changes in the nature of the fats consumed, which, in the US, ranged from less than 5% of total fatty acids in the 1950s to more than 15–25% today [28].

Dovetailing on the seminal study, other studies have found that breast milk composition is determined by the maternal diet [29,30], and suggest that the high presence of LA in breast milk primarily indicates the pervasiveness of LA in the food supply. An extensive body of literature on human breast milk fatty acids from different countries in Africa and South America also shows that the LA content in breast milk varies widely depending on maternal lipid nutrition [31,32].

Breast milk LA is also derived from the mobilization of adipose tissues, which have been partly constituted before and during pregnancy [33]. Thus, while LA provides nutritive value to infants in reasonable amounts, the contributions of both maternal diet and maternal adipose tissues seem to underlie the varying LA contents in breast milk noted throughout the world.

## 4. Historical Intake Patterns and Current Sources

Prior to the 20th century, the average intake of LA was under 2% of the total daily caloric intake. The biological optimal range is approximately 1% to 2%, but current LA consumption is over 25% of the total calorie intake for the average person [34]. The consumption of LA at these levels lowers the metabolic rate [35,36] and increases tissue oxidative damage that increases susceptibility to chronic diseases. Consistently elevated LA intake likely accelerates the biological clock, resulting in premature aging and death [37].

Historically, LA intake increased from approximately 2 g/day in 1865 to 5 g/day in 1909, followed by 18 g/day in 1999, and more recently up to 29 g/day in 2008. LA consumption accounted for approximately just 1/100th (1%) of the total caloric intake in 1865, with an observed increase of more than one-fourth of the total calories by 2010, reflecting a 25-fold increase [38].

### LA Consumption of Seed Oils in the US

Before 1866, the Western diet consisted mainly of animal fats, such as tallow (beef fat), suet (mutton, beef, or lamb fat), lard (pork fat), and butter (milk fat) [39]. Furthermore, Eastern societies used cold-pressed fats, such as coconut and palm oil. Vegetable and seed oils that are regularly consumed today did not exist prior to the late 1800s.

A fundamental change in agricultural history was the shift from the extraction of cold-pressed plant and seed oils to industrially processed seed oils after the US Civil War [40,41]. However, the use of this new innovation did not gain in popularity quickly, even with tactical marketing strategies. By the mid-1900s, animal foods still provided 99% of added fats in the human diet, but 86% of added fats came from seed oils by 2005.

Currently, the global consumption of seed oils is approximately 200 million tons per year, with a projected rate of 258.4 million tons by 2026 [42]. Today, the majority of ultra processed foods contain one or more forms of industrially processed seed oils, including potato chips, cookies, pastries, and bread. Less obvious and stealthy sources include most food establishments (e.g., restaurants), as they primarily use processed seed oils to prepare their food.

The primary current source of long-chain fatty acids are omega-3 oils (e.g., EPA and DHA) from seafood (fish), crustaceans (lobsters), and mollusks (squid and oysters). Some land animals, such as grass-fed beef, also provide long-chain omega-3 fatty acids in smaller quantities than marine sources [43]. Plant-based sources, such as canola, linseed, soybean, and cottonseed, contain small amounts of omega-3 fatty acids, but these are not the beneficial long-chain varieties.

Furthermore, short-chain omega-3 fatty acids in plants and seeds do not confer the same health benefits as long-chain omega-3 fatty acids, mainly due to their LA component that leads to health problems when this PUFA begins to accumulate in the blood and human tissues [44].

## 5. The Omega 3:6 Ratio

Among the many types of omega-3 fatty acids, the three most important are eicosapentaenoic acid (EPA), docosahexaenoic acid (DHA), which are “long-chain” omega-3 fatty acids, and alpha linoleic acid (ALA)—a “short-chain” omega-3 fatty acid. The human body is unable to produce essential fatty acids endogenously.

Therefore, omega-3 fatty acids must be consumed regularly through the diet. Animal foods such as cold-water fish are ideal sources of EPA and DHA, while ALA is mainly derived from plants. EPA and DHA have anti-inflammatory effects and ALA must be converted to EPH and DHA. This conversion is an inefficient process, particularly in men, with less than 20% being converted [45]. Therefore, special attention should be paid to obtaining EPA and DHA through diet or supplementation.

The benefits of maintaining the proper omega 3:6 ratio are well-established. Bodily tissues consist primarily of saturated and monounsaturated fats, which are a readily utilized source of nutrients that support the development and maintenance of cells [46]. The primary dietary PUFAs are omega-3 and omega-6 fats, which the body needs in relatively small quantities.

It is important to consume sufficient amounts of omega-3 fatty acids to sustain optimal health, with the recommended daily serving being between 500 and 1000 milligrams of omega-3 fatty acids [47,48]. Contrary to previous perceptions, however, consuming larger servings of omega-3 fatty acids does not support an ideal ratio. Instead, excessive quantities of omega-3s may cause additional metabolic damage—similar to that which occurs due to the conversion of elevated LA levels.

Furthermore, the circulation of excessive LA in the bloodstream, regardless of omega-3 fatty acid intake, contributes to the pathology of diseases. A more useful approach to increasing omega-3 fatty acid levels in the body and maintaining a healthy omega 3:6 ratio is by decreasing the consumption of omega-6 fatty acids, including LA [49].

One reason omega-3 fatty acids are particularly beneficial is due to their anti-inflammatory properties, especially that which is derived from animal sources [50]. DHA in bodily tissues is a precursor for the synthesis of compounds called resolvins, which help reduce inflammation by heightening the macrophage phagocytosis of debris and counteracting proinflammatory molecules [51].

Similarly, diets that are rich in whole foods, as opposed to synthetically derived foods, have been shown to reinforce cardiovascular health by lowering lipid and triglyceride levels in the blood, decreasing blood viscosity, reducing platelet aggregation (clot formation), and lowering the risk of heart attack [52,53].

## 6. Pathophysiological Mechanism of Elevated LA Levels

**Figure 1:**
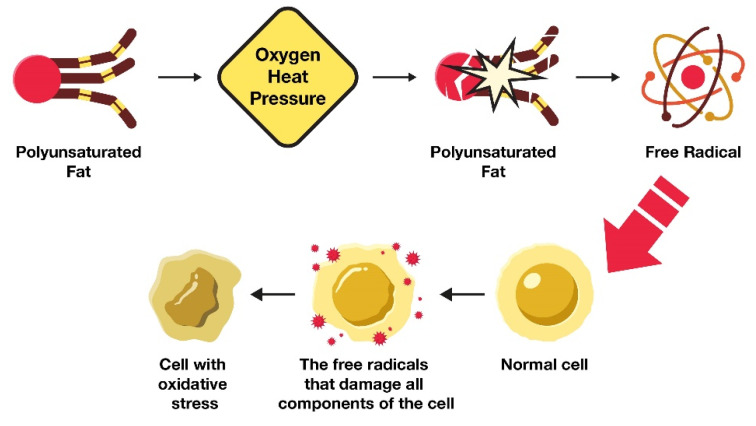
Mechanism of oxidative stress generated through oxidation of polyunsaturated fats.

As revealed in Figure 1, the primary reason why excess LA in the body leads to cellular tissue damage is because this highly fragile PUFA is easily altered through oxidation. Like other types of PUFAs, LA consists of fragile double bonds that are susceptible to oxidative damage [54].

The double bonds are the key to understanding why PUFAs are highly perishable and prone to oxidation. LA is metabolized in a multistep pathway to arachidonic acid (AA) through the conversion to gamma-linolenic acid (GLA) due to Δ^6^desaturase enzyme, which varies based on the activity of desaturase enzymes. GLA is converted to dihomo-γ-linolenic acid (DGLA), which is the immediate precursor to AA. Chronically elevated levels of AA can contribute to a state of chronic inflammation and have been associated with autoimmunity [55]. This has led to some popular debate regarding the influence of LA intake on AA levels, with some recent papers suggesting that an excessive LA intake can lead to the accumulation of AA-derived pronociceptive lipid mediators and a reduction in antinociceptive lipid mediators obtained from omega-3 fatty acids [56,57]. However, a systematic review found that LA intake was not associated with tissue concentrations of AA in human beings [58]. Thus, the conversion of LA to AA is unlikely to be a concern to human beings at modest levels of consumption.

However, during metabolic processes (e.g., energy production or utilization), the LA double bonds become vulnerable to damage by oxygen, heat, and pressure. When they become damaged or oxidized, they are converted into harmful metabolites (e.g., OXLAMs and OXAAMs). It is these metabolites, and not the PUFAs themselves, that are responsible for their profoundly negative impact on health.

### 6.1. How Excess LA Consumption Alters Health

As noted previously, the conversion of LA to AA is unlikely to play a major role in the pathology related to excessive LA intake. LA’s well-documented propensity to enhance lipid peroxidation and the formation of oxidized LA-derived lipid metabolites (OXLAMs) is more likely to contribute significantly to its detrimental effects.

Lipid peroxidation is a well-established process associated with various chronic diseases. Excessive iron, for example, contributes to this damage through a process called ferroptosis, characterized by the accumulation of iron-dependent lipid peroxides that cause membrane injury and cell death. This mechanism plays a significant role in the development of cancer cardiovascular diseases, neurodegeneration and aging.

Membrane lipids containing PUFAs, such as LA, are particularly susceptible to peroxidation due to the weak C-H bonds between adjacent C=C double bonds. During lipid peroxidation, primary products such as lipid hydroperoxides are formed, which can further oxidize to produce reactive aldehydes, with 4-hydroxynonenal (4-HNE) being the most extensively studied and biologically relevant product of LA peroxidation. The accumulation of peroxidized lipids, including 4-HNE, can influence cellular processes, ranging from proliferation to apoptosis and necrosis, depending on their concentration.

4-hydroxynonenal (4-HNE) is an extraordinarily reactive compound and one of the most studied and seemingly biologically relevant products of lipid peroxidation, and is a product of LA peroxidation. Peroxidized lipids are bioactive. They can have effects on cells similar to those of hydrogen peroxide: low levels can stimulate proliferation; higher levels block proliferation and yet higher ones induce apoptosis and necrosis. 4-HNE tends to concentrate in biomembranes rather than in the aqueous space of cells.

When one has elevated tissue levels of LA, consuming an excess of LA, even from unprocessed whole foods, can accelerate metabolic damage. This is due to the propensity for LA to be converted to OXLAMs or OXAAMs that can damage structures, including DNA, mitochondria, cell membranes, proteins, and stem cells [59,60,61].

Another problem with PUFAs, such as LA, is that they are chemically unstable, and it is well known that energy metabolism is linked to the conversion of excess LA to oxidant species [62]. These highly oxidatively reactive metabolites contribute to most of the oxidative damage observed in pathological conditions.

Metabolites that are generated following the consumption of processed seed oils have been associated with mitochondrial dysfunction [63,64,65,66,67], abnormal levels of inflammation, and endothelial cell damage [68]. The formation of OXLAMs has also been linked to memory impairments and an increased risk of Alzheimer’s disease. Canola oil, in particular, has been linked to Alzheimer’s [69].

In addition, oxidized metabolites deplete glutathione levels in the liver, thus, lowering antioxidant defenses, impairing immune function, and increasing mortality [70]. Furthermore, oxidized species lead to fat cell insulin resistance [71], as well as the inhibition of cardiolipin [72]—an important fat located in the inner membrane of mitochondria.

Additionally, the body has enzymes called delta-desaturases and elongases that convert short-chain omega-3 fatty acids—such as ALA, which is typically found in plants (e.g., chia seeds, flax seeds, and walnuts)—to long-chain fatty acids (e.g., DHA and EPA) [73]. The human body converts short-chain omega-3 fatty acids from plant-based sources to long-chain fatty acids with an average efficiency of approximately 5% [74].

The body utilizes long-chain fatty acids that are typically found in animal foods more efficiently (e.g., cold-water fish and grass-fed beef), as these sources provide sufficient amounts of DHA and EPA. Certain macroalgae (e.g., seaweed and nori) and microalgae species (e.g., spirulina and chlorella) also produce high levels of EPA and DHA [75].

However, when large amounts of LA are consumed through the diet, delta desaturase enzyme activity is inhibited, making it difficult for the body to convert short-chain omega-3 fatty acids, like ALA, into long-chain omega-3 fatty acids [76,77,78]. This process further increases the dependence on animal foods or edible algae as a source of EPA and DHA.

### 6.2. LA Remains in Tissues for Extended Time Periods

Another major reason why seed oils are pernicious to overall health is that they remain in the body for extended periods. The half-life of LA is approximately 680 days, or approximately two years [79]. This means that it takes approximately six years to replace 95% of the LA in the body with healthy fats—making this a primary reason to maintain low LA intake. Omega-3 fatty acids, such as DHA and EPA, have half-lives of 2.5 years and a few months, respectively [80,81]. The body also converts certain amounts of DHA to EPA.

Furthermore, the refined sugars contained in many ultra processed foods are rarely present without seed oils. The body can only store a limited amount of carbohydrates in the form of sugar called glycogen that is primarily stored in the liver and muscles. In the absence of an adequate sugar and carbohydrate intake, most individuals exhaust their glycogen stores within several days. Consuming large amounts of added sugar, while not ideal, does not lead to storage for years. This is in contrast to LA derived from seed oils, which remains in the body for up to six years, thereby contributing to many chronic degenerative diseases [82].

### 6.3. Cardiolipin: Stealth Fat in Mitochondria

Mitochondria are subcellular organelles that are responsible for producing most of the body’s cellular energy in the form of adenosine triphosphate (ATP) [83]. It is the presence of mitochondria that distinguishes mammals (e.g., humans) from bacteria and allow life to be multicellular. 

These organelles produce approximately 85% of the energy for the body through the generation of ATP during oxidative phosphorylation. If mitochondrial dysfunction develops, physical symptoms (e.g., chronic fatigue) may arise, along with an increased susceptibility to disease. It is vital to take preventive measures to improve and maintain mitochondrial health as it profoundly impacts longevity. Cardiolipin optimization reinforces enhanced mitochondria activity and energy production.

**Figure 2:**
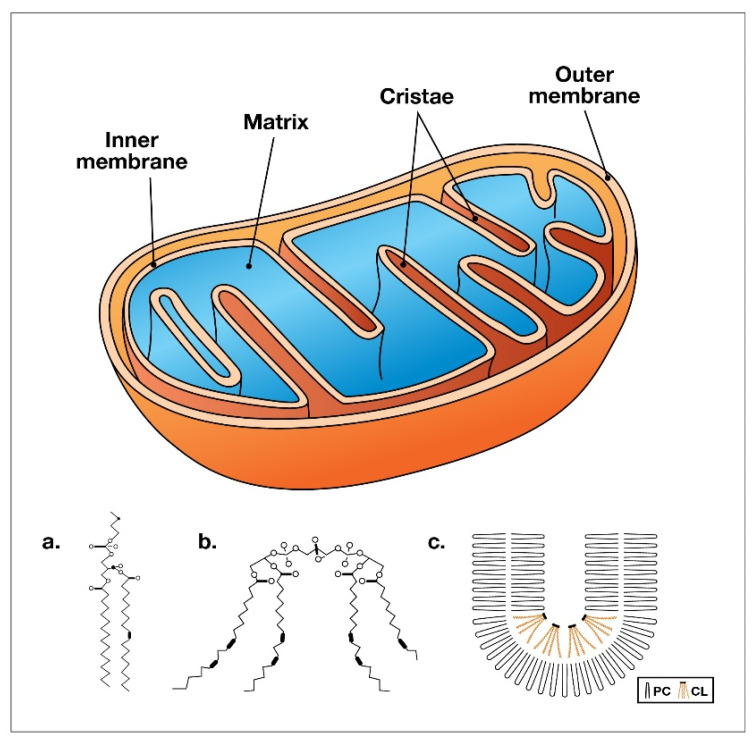
Mitochondrion structure (left): (**a**) molecular diagram of cardiolipin (CL); (**b**) with the four fatty acids; (**c**) folding pattern of phosphatidylcholine (PC) and CL that provides the curve in the mitochondrial cristae [84].

At the molecular level, excess LA consumption damages mitochondrial metabolism and impedes the body’s ability to generate ATP. Cardiolipin is a phospholipid that is only located in mitochondria, with the highest levels being localized in the inner mitochondrial membrane, as illustrated in Figure 2.

To emphasize the importance of this phospholipid, 20% of the fat in the mitochondria is found in the form of cardiolipin [85]. The human body has over 100,000 trillion mitochondria, and mitochondrial health is largely dependent upon the type of dietary fats that are available for cardiolipin synthesis within these specialized organelles.

Cardiolipin is composed of four fatty acids [86], unlike triglycerides, which have three fatty acids, but the individual fats that comprise cardiolipin vary widely. Examples include LA, palmitic acid, and the fatty acids found in fish oil, such as DHA and EPA. Cardiolipin is synthesized from fatty acids that are consumed through the diet; thus, the overconsumption of LA in the form of seed oils can alter the formation of the inner mitochondrial membrane, cristae, and complex IV. 

Each of these fatty acids have a different effect on mitochondrial function, and the impact of each distinct fatty acid type also depends on the organ in which mitochondria are located. Figure 1 illustrates a typical mitochondrion, demonstrating how the folding pattern of cardiolipin leads to the curved formation of the mitochondrial cristae. The folding causes the super complexes in the electron transport chain to come closer together and more efficiently transfer electrons that ultimately result in the production of ATP.

Cardiolipin molecules containing one or more LA fatty acids are highly susceptible to free-radical-induced lipid peroxidation. The oxidation of cardiolipin is involved in regulating apoptosis, mitophagy, and other cellular functions. Interestingly, LA-containing cardiolipin is preferentially oxidized over other phospholipids in the inner mitochondrial membrane, even in the presence of more-oxidizable fatty acids such as arachidonic acid.

This is important, because mitochondrial lipids are necessary for maintaining the structural integrity and proper functioning of mitochondria. Cardiolipin is prone to free-radical-induced lipid peroxidation due to the presence of up to four chains of LA. The oxidation of cardiolipin plays an important role in the regulation of apoptosis, mitophagy, and other cellular functions.

The oxidation of cardiolipin plays an important role in the regulation of apoptosis, mitophagy, and other cellular functions. LA-containing cardiolipin is preferentially oxidized over other phospholipids that are found in the inner mitochondrial membrane, including phosphatidylinositol, phosphatidylserine, phosphatidylinositol, and phosphatidylethanolamine. This occurs even in the presence of more oxidizable fatty acids, such as arachidonic acid.

Emerging evidence suggests that mitochondrial lipid peroxidation not only affects the structural integrity of mitochondria, but also mitochondrial functions, such as protein transportation, respiratory metabolism for ATP generation, mitochondrial dynamics and quality control through the fission and fusion of mitochondria, and mitophagy.

Mitochondrial proteins, which constitute a significant portion of proteins within the inner mitochondrial membrane, are highly susceptible to modification by 4-HNE. Approximately thirty percent of all proteins modified by 4-HNE are mitochondrial proteins. Consequently, endogenously produced 4-HNE resulting from oxidative stress has been shown to cause mitochondrial dysfunction in various cell types and organs during both physiological and pathological conditions. This vulnerability of mitochondrial proteins to 4-HNE modification contributes to their critical role in the development of mitochondrial dysfunction.

Research shows that the dietary fats involved in cardiolipin synthesis are directly regulated by the types of fats that are consumed in the diet [87]. LA is particularly susceptible to oxidation when it is embedded within a cardiolipin molecule. The heart preferentially accumulates cardiolipin-containing LA, while the brain preferentially accumulates cardiolipin derived from DHA. Changes in fatty acid intake that include avoiding sources of LA (e.g., industrially processed seed oils) may gradually improve cardiolipin composition throughout the body and reinforce long-term health. Further prospective studies are needed to evaluate this hypothesis.

## 7. Associations between LA Intake and Chronic Disease

Oxidative stress, tissue damage, and mitochondrial dysfunction from excess LA is not only responsible for cardiovascular disease and Alzheimer’s onset, but additional chronic diseases, including cancer, dementia, obesity, and diabetes are also associated with oxidized metabolites. There is conflicting evidence on the associations between LA intake and many of these chronic diseases, which are summarized below in Table 1.

While the evidence is still conflicting in some instances, the mechanisms underlying excessive LA intake are reflected in a wide variety of chronic diseases. A recent study [99] found that high-fat diets increased the formation of vitamin A degradation products, known as bisretinoids, in ocular tissues. These degradation products are known to cause damage to the retina directly, but they also participate in the formation of lipofuscin in the retina. Lipofuscin is a byproduct of PUFA peroxidation. The study found the PUFA linoleic acid to be a causal factor of eye damage.

Soy oil is by far the most widely produced and consumed seed oil in the US. Using mice, researchers in 2020 found that a high soybean oil diet not only led to obesity and diabetes, but could also affect neurological conditions such as autism, Alzheimer’s disease, anxiety, and depression [100]. The same research team found in 2015 [101] that soy oil induced obesity, diabetes, insulin resistance, and fatty liver in mice. Then, in a 2017 study [102], the same group learned that if soy oil was engineered to be low in LA, it induced less obesity and insulin resistance.

Type I diabetes is an autoimmune condition. The beta-cells of the pancreas are attacked by antibodies from the immune system, over time leading to their destruction and the inability to produce insulin. One study [103] found that the reason for antibody production was the overexpression of the enzyme 12/15-lipoxygenase, which is an enzyme involved not only in synthesizing inflammatory leukotrienes, but also metastatic cancer.

Leukotrienes are PUFA metabolites and are responsible for the development of type I diabetes. This suggests that a 12/15-LOX inhibitor would be therapeutic. This study administered a leukotriene inhibitor and found it effective in preventing the development of beta-cell autoimmunity. A more fundamental approach would be to avoid or replace the source of leukotrienes, which would be dietary PUFAs.

### 7.1. Obesity and LA

In the US, nearly 43% of adults 20 years of age and older are obese [104], while approximately 74% of all adults are overweight or obese [105]. Although these statistics are alarming, the American Obesity Association suggests that by 2025, 50% of Americans may be obese. Predictions also indicate that the percentage is likely to rise to 60% by 2030 [106]. There is a growing body of evidence based on animal studies suggesting that polyunsaturated fatty acids (PUFAs), such as those found in vegetable oils, contribute to the obesity epidemic [107,108,109,110].

**Figure 3:**
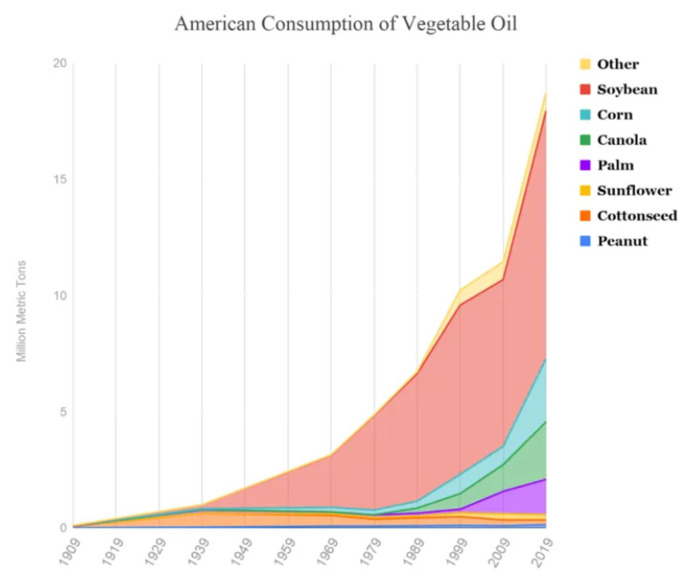
Change in American consumption of vegetable oils between 1909 and 2019.

It is also important to note that the US has both the highest obesity rate in the developed world [111] and the highest consumption of seed oils per person than any other nation [112]. Figure 3 illustrates the ongoing shift in vegetable oil consumption that began in the early 20th century, as industrially processed vegetable and seed oils entered the food supply and gradually displaced natural animal fats.

**Figure 4:**
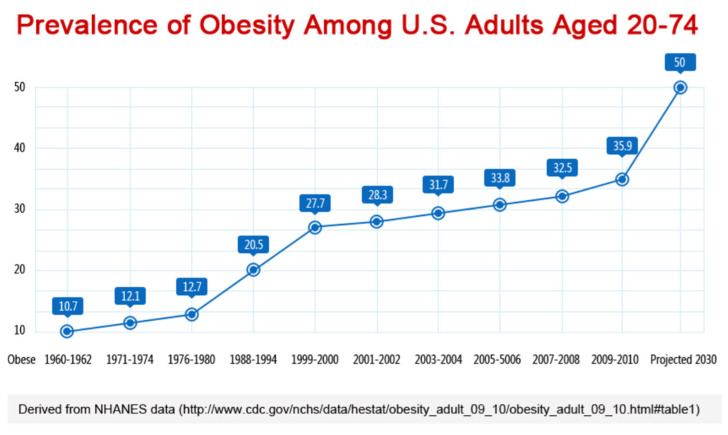
Data from the Centers for Disease Control and Prevention (CDC).

Figure 4 also demonstrates the gradual rise in obesity rates for American adults that coincided with the increased intake of vegetable and seed oils, the main sources of elevated LA intake. A continuous increase in obesity prevalence has been projected for the year 2030, emphasizing the importance of evaluating approaches to reducing LA consumption and demonstrating the rise in obesity rates for adolescents and adults, along with the projected prevalence of obesity by 2030.

While there appears to be a coincident rise in both the consumption of LA-containing seed oils and obesity, it should be noted that this does not imply causality. There are a number of other dietary (e.g., refined sugars, ultraprocessed foods, etc.) and nondietary (e.g., physical inactivity, environmental toxicants, poor sleep, etc.) risk factors that have also increased during this time. Nonetheless, LA is one such risk factor worthy of consideration.

### 7.2. Cardiovascular Disease and LA

In the 19th century, a cardiovascular disease diagnosis was rare [113,114], and there were only nine papers reported in the literature that documented cardiovascular disease. Furthermore, the first heart attack reported in the US was in 1912 [115], and it was not until 1920 that this disease became more prevalent, leading to the establishment of the American Heart Association (AHA) [116].

One of the first changes that occurs in atherosclerosis, which is the precursor to cardiovascular disease, is the alteration of macrophages. Under atherosclerotic states, macrophages transform into foam cells, which is essentially a macrophage that is embedded with fat and cholesterol. Therefore, atherosclerotic plaque comprises dead macrophages and other types of cells with the accumulation of cholesterol and fat.

This is one of the main reasons cardiovascular disease is often linked to dietary cholesterol (low-density lipoprotein (LDL)) and fat. However, researchers have observed that for foam cells to form, LDL cholesterol must be modified through oxidation, and this occurs when excessive seed oils are consumed. Industrially processed seed oils cause LDL to oxidize, thereby promoting the formation of foam cells.

LA has been recommended by many professionals to lower the risk of heart disease, as it lowers LDL. This is related to a widespread misconception that atherosclerotic plaque is caused by high concentrations of LDL and cholesterol in the blood. However, research suggests that the mechanism driving atherosclerosis appears to be the oxidation of PUFAs, specifically LA, in the LDL membrane. This is because excess PUFAs lead to fragile cell membranes that can be easily damaged by oxidation [117,118].

This observation is also supported by Ramsden’s reanalysis of the data from the Sydney Diet Heart Study, a randomized controlled trial that investigated the effects of a dietary intervention low in saturated fat and high in PUFAs (including LA) on the incidence of heart disease. This study found that the intervention group had a significantly higher risk of all-cause mortality and cardiovascular disease mortality as compared to the control group [119].

Wu et al. analyzed data from the Cardiovascular Health Study, a prospective cohort study of older adults in the US, and found that higher levels of LA in the blood were associated with a higher risk of all-cause mortality as well as cardiovascular disease and cancer mortality [120]. Li et al. analyzed data from the Nurses’ Health Study and the Health Professionals Follow-up Study. These two large prospective cohort studies were conducted in the US and found that a higher intake of LA was associated with a higher risk of coronary heart disease mortality [121].

This also means that when conventional treatment methods are recommended such as statin drugs—which effectively reduce plaque buildup, but do not address the root problem—the body is not able to properly manage LDL and high-density lipoprotein (HDL) levels. Over time, this may still lead to an increased risk of heart failure. Research has also shown that oxidized LDL is a better predictor of cardiovascular disease than LDL [122,123]. Thus, LDL does not appear to initiate atherosclerosis, and LDL’s susceptibility to this oxidative process is controlled by LA intake from dietary sources.

In 1961, the first recommendations were released by the AHA, urging the reduced intake of saturated fats and cholesterol from animal sources and to replace such fats with PUFAs, such as seed oils [85,86]. This advice was adapted in 1977 for the US Dietary Guidelines for Americans. As a result, industrially processed seed oils were marketed as health foods and their consumption gradually increased. In addition, public health officials disparaged animal fats, resulting in a nearly uniform negative public perception of saturated fats.

Other organizations also began to promote the use of PUFA-based seed oils to remove and discredit the remaining competitors of seed oil, such as cold-pressed palm and coconut oil. Prior to this movement, the American Soy Association successfully lobbied Congress in the 1930s to enforce taxes on Asian-made cooking oils and fats [124].

Furthermore, the American Fats and Oils Association also initiated discrediting campaigns when cold-pressed oils started to regain popularity in the 1980s. At the time, people in Malaysia and the Philippines consumed large amounts of cold-pressed palm and coconut oil, and the incidence of cardiovascular disease there was very low.

Despite evidence of the health benefits associated with cold-pressed tropical cooking oils and saturated fat, Americans have been targeted with discrediting propaganda against saturated animal fats for the last 75 years [125,126]. Although conventional medicine has advised the public to replace saturated fats from animal foods with processed seed oils to lower the risk of cardiovascular disease, human trials have demonstrated that industrially processed seed oils do not decrease atherosclerosis rates or the risk of dying from cardiovascular disease [127].

The primary reason for atherosclerotic changes involves OXLAMs [128]. One type of OXLAM is 4-hydroxynonenal (HNE), a mutagen and biomarker of oxidative stress that is known to cause DNA damage [129]. The levels of 4-HNE are relatively easy to measure and studies have shown associations between elevated 4-HNE levels and heart failure [130,131]. It is important to note that LA is broken down into 4-HNE faster when the seed oil is heated, which is why most cardiologists recommend avoiding fried foods.

### 7.3. Cancer and LA

Cardiovascular disease is not the only condition triggered by excessive LA intake and the subsequent OXLAMs produced. Oxidized metabolites also play a significant role in cancer, which is the second-leading cause of death in the US [132]. Animal models have demonstrated a link between an increased incidence of cancer following seed oil consumption [133], with animals typically developing cancer once the LA intake in their diet reaches 4% to 10% of their energy intake.

Furthermore, research shows an association between LA intake in the form of omega-6 fatty acids and in increased risk of developing skin cancers [134], and there is also evidence that shows that eliminating seed oils from the diet can dramatically reduce the risk of ultraviolet (UV)-induced sunburn [135]. Susceptibility to UV radiation damage of the skin is directly influenced by the amount of LA in the diet [136,137].

PUFAs can also function as a cancer “sensitizer” and trigger the development of cancer in the presence of the pervasive synthetic chemicals present in the environment that are relatively harmless by themselves. One study demonstrated that dietary PUFAs transform even very low doses of heterocyclic amines from almost completely harmless to strongly carcinogenic [138].

## 8. Dietary Sources of LA and Mitigation Strategies

Table 2 below provides a comprehensive list of the most consumed edible oils and their approximate LA content [139,140,141]. In general, the lowest LA-containing source of fats would be the preferred fats of choice for lowering the LA burden in the diet. Olive oil is a popular cooking oil that is prominently featured in Mediterranean diets, which generally contain far fewer seed oils considering the abundant use of olive oil. 

However, olive oil demonstrated a nearly 10-fold wide variability in the percentage of LA and the vast majority of commercial olive oil, and avocado oils, are adulterated with seed oils. A recent study evaluated 89 olive cultivars and found a range of 3% to 27% levels of LA [142]. Tests have also revealed that anywhere from 60 to 90% of the olive oils sold in American grocery stores and restaurants are adulterated with cheap, oxidized, omega-6 vegetable oils, such as sunflower oil or peanut oil, or nonhuman-grade olive oils, which are harmful to health in a number of ways [143].

Although this problem is concerning, instead of avoiding all cooking oils (e.g., vegetable and seed oil), healthier choices include those that have been used for centuries, such as butter and beef tallow. In addition to containing the lowest LA content, these sources of fats also provide the fat-soluble vitamins A, D, and K_2_.

Despite these sources being readily available, most Americans simply fail to obtain enough preformed vitamin A in their diet. This can contribute to many chronic diseases, including cardiovascular disease [144] and cancers [145]. An elevated seed oil intake also adversely impacts health, survival, and vision [146]. Coconut oil is also very low in LA but does not have the essential fat-soluble vitamins that tallow and butter contain.

Nuts and seeds are often promoted as being ‘heart healthy’ [147]. However, Table 3 shows that the LA content of most nuts and seeds is exceedingly high in LA. For example, pecans consist of 50% LA [148]. The composition is similar to the amount of LA in many seed oils. The only exception is macadamia nuts.

Furthermore, while nuts and seeds are often consumed in unprocessed form and do not contain many advanced lipoxidation end-products (ALEs) such as 4-HNE, unlike high PUFA seed oils, they still increase the LA content of the diet. This means that even though nuts and seeds are among the best types of omega-6 fats to consume, it may be advisable to limit consumption unless one is eating less than 5 g of LA per day. 

Nuts and seeds are likely not a problem as long as the total daily intake of LA is below 2% of total daily calories. It is important to note that only 2% of the fat in macadamia nuts is in the form of LA, which means that macadamia nuts can be consumed without raising LA levels. However, it is still typically recommended to limit the intake of macadamia nuts to a few ounces a day or less.

### 8.1. Sources of Animal Protein and Varying LA Contents

Animals are typically fed corn, soy, or other seeds and grains, which is radically different from their native, traditional, or ancestral diet. This presents a problem for nonruminant animals due to the concentration of LA in the seeds and grains they are fed. Ruminants are animals with multiple parts to their stomachs. This includes cows, buffalo, sheep, lamb, goats, deer, elk, and many other game animals.

Ruminants have low LA in both their meat and milk, no matter what they eat [149]. This is because their stomach has a ‘biohydrogenation chamber’ that contains bacteria that can convert the high LA fat they eat from grains and seeds into saturated and monounsaturated fats. This is in contrast to animals with one stomach, such as chickens and pigs, that when fed a diet high in LA, including corn and soy, experience an increase in high levels of LA in their tissues, similar to the process that has been observed in humans [150].

The ability of the biohydrogenation chamber to efficiently convert high LA fat to saturated and monounsaturated fats is well known, as the difference in LA in ruminants that are 100% grass-fed and those that are fed corn and soy is only approximately 0.5%. This is why, from an LA intake perspective, there is not much difference between concentrated animal feeding operation (CAFO) beef and grass-fed-only beef. However, grass-fed beef is preferred, as it contains less glyphosate, other toxins, and hormones.

Consuming animal protein from ruminants is optimal and, ideally, the protein source should be organic. Furthermore, the animals should not be fed any food that is contaminated with glyphosate, hormones, or other pesticides. It is also important to reduce the intake of chicken or porcine (pig) meat, as nearly all conventionally raised chickens and pigs are fed soy and corn, typically genetically modified organism (GMO) varieties that are sprayed with glyphosate.

Even if chickens and pigs are fed organic soy and corn, they typically contain substantial amounts of LA, resulting in most chicken and porcine (pig) meat containing over 25% LA. Chicken eggs, as opposed to the meat, are acceptable, as each egg contains less than 1 g of LA, assuming they are not fed commercial feeds with high concentrations of LA. Chickens can also be farm-raised or free range to control their feed intake and reduce the high LA content.

### 8.2. Carnosine Helps Lower Oxidative Damage from LA

**Figure 5:**
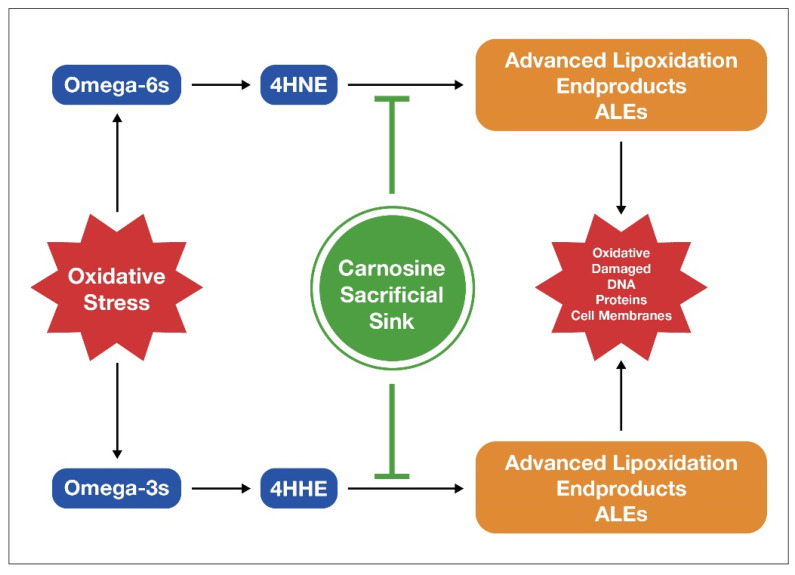
Carnosine scavenges ROS and ALES such as 4-HNE generated through the oxidation of fatty acid cell membranes during oxidative stress.

Carnosine is an endogenously produced dipeptide, and it consists of only two amino acids, beta-alanine and histidine [151]. It is a potent antioxidant that helps limit the damage from excess LA by binding to ALEs. It serves as a sacrificial sink for reactive oxygen species (ROS) and ALEs [152] by letting these damaging molecules destroy it rather than mitochondria, DNA, or proteins, as depicted in Figure 5.

The highest concentrations of carnosine are found in the muscles and brain [153]. Carnosine is also found in meats but is not contained in any plant foods. That is why individuals following a vegetarian or vegan diet typically have lower levels of carnosine in their muscles. This is also one reason why many strict vegans who do not properly compensate for low carnosine intake from meat and other nutritional deficiencies may experience difficulties building muscle.

However, carnosine itself may not be as useful as a dietary supplement, as it is rapidly broken down into its constituent amino acids such as beta alanine and histidine by certain enzymes. The body then reformulates these amino acids back into carnosine that is stored in muscle tissue.

Furthermore, consuming carnosine as a supplement is more expensive and it is not as efficient as taking beta-alanine. A far more efficient alternative is to supplement the diet with beta-alanine, which appears to be the rate-limiting amino acid in the formation of carnosine. Eating animal protein is also known to efficiently raise carnosine levels in the muscle [154], which is why beta-alanine supplementation may be particularly important for vegetarians or vegans.

## 9. Limitations

There are several key limitations to this review that are worthy of mention. While the intention of this narrative review was to provide a tempus excursus of linoleic acid intake in the standard American diet and the related mechanisms that may contribute to the rise in chronic disease over time, a more formal systematic review of the individual diseases would add further precision and replicability to the associations described in this paper. In addition, many of the studies used to both classify LA as an essential fatty acid and to mitigate its deleterious effects on health were performed in animal models. As such, more mechanistic studies in humans are needed.

## 10. Conclusions

The dramatic increase in LA intake in the standard American diet appears to contribute to the simultaneous rise in a wide variety of chronic diseases. While modest amounts of LA support human health, deleterious mechanisms of excessive LA intake include the formation of oxidized linoleic acid metabolites (OXLAMs) and a suboptimal cardiolipin composition. These disruptions to optimal physiology cause impairments in mitochondrial function, compromised metabolic function, and excessive inflammation, all of which contribute to obesity, cardiovascular disease, cancer, and many other chronic conditions that plague our healthcare system.

While other dietary factors, such as refined sugars and ultraprocessed foods, more generally, contribute to the rise in chronic diseases, the long half-life of LA and its integration into cardiolipin with excessive intake is particularly pernicious. Future prospective studies of low-LA diets in humans appear warranted and should consider the relatively long timeframe for the reduction in the harms of an excessive LA intake. Furthermore, while the standard American diet has become more pervasive worldwide, including in China [57.89] and India, the hypothesis that excessive LA intake may be contributing to chronic disease explored in this review should be evaluated more closely in populations outside of the United States.

## Figures and Tables

**Table 1:** Summary of health effects of linoleic acid.

Proposed Neutral or Health Benefits	Health Risks
Reduces cardiovascular disease risk by decreasing total cholesterol levels [88]	Increases risk of cardiovascular disease by increasing oxidized LDL [2,89]
Unrecognized as having an impact on cancer [90]	Increases risk of cancer by impairing mitochondrial function and increasing systemic oxidative stress [91] that adversely impacts cardiolipin in the inner mitochondrial membrane [92,93,94]
Reduces the risk of type 2 diabetes [95]	Increases risk of diabetes [91]
Role in obesity is contentious [96]	Increases risk of obesity [97]
Unrecognized as having an impact on dementia	Increases risk of dementia [98]

**Table 2:** Most commonly consumed cooking oils and percentages of LA content.

COOKING OILS	% LINOLEIC ACID (LA) AVERAGE VALUE (RANGE IN PARENTHESES)
SAFFLOWER OIL	70%
GRAPE SEED OIL	70%
SUNFLOWER OIL	68%
CORN OIL	54%
COTTONSEED OIL	52%
SOYBEAN OIL	51%
RICE BRAN OIL	33%
PEANUT OIL	32%
CANOLA OIL	19%
OLIVE OIL	10% (3–27%)
AVOCADO OIL	10%
LARD	10%
PALM OIL	10%
TALLOW (CAFO)	3%
GHEE/BUTTER (CAFO)	2%
COCONUT OIL	2%
TALLOW (GRASS FED)	1%
BUTTER (GRASS FED)	1%

Red—high linoleic acid; yellow—moderate linoleic acid; green—low linoleic acid.

**Table 3:** Commonly consumed seeds and nuts and percentages of LA content.

SEEDS/NUTS	% LINOLEIC ACID (LA) AVERAGE VALUE (RANGE IN PARENTHESES)
POPPY SEED	62%
HEMIP	57%
WHEAT GERM	55%
WALNUT	53%
PECAN	50%
PUMPKIN	45%
BRAZIL NUTS	43%
SESAME	41%
PEANUT	32%
PINE NUTS	33%
CHIA	16%
ALMOND	16%
FLAXSEED	14%
PISTACHIO	13%
HAZEL NUTS	12%
CASHEW	8%
MACADAMIA	2%

Red—high linoleic acid; yellow—moderate linoleic acid; green—low linoleic acid.

## Data Availability

Not applicable.
